# Prosocial Behavior Is Associated With Transdiagnostic Markers of Affective Sensitivity in Multiple Domains

**DOI:** 10.1037/emo0000813

**Published:** 2020-07-27

**Authors:** Luis Sebastian Contreras-Huerta, Patricia L. Lockwood, Geoffrey Bird, Matthew A. J. Apps, Molly J. Crockett

**Affiliations:** 1 Department of Experimental Psychology and Wellcome Centre for Integrative Neuroimaging, University of Oxford, and Centre for Human Brain Health, School of Psychology, University of Birmingham, UK; 2 Department of Experimental Psychology, University of Oxford; 3 Department of Experimental Psychology and Wellcome Centre for Integrative Neuroimaging, University of Oxford, and Centre for Human Brain Health, School of Psychology, University of Birmingham, UK; 4 Department of Experimental Psychology, University of Oxford, and Department of Psychology, Yale University

**Keywords:** prosocial behavior, affective traits, psychiatric traits, transdiagnostic, computational modeling

## Abstract

Prosocial behaviors—actions that benefit others—fundamentally shape our interpersonal interactions. Psychiatric disorders have been suggested to be related to prosocial disturbances, which may underlie many of their social impairments. However, broader affective traits, present to different degrees in both psychiatric and healthy populations, also have been linked to variability in prosociality. Therefore, it is unclear to what extent prosocial variability is explained by specific psychiatric disorders relative to broad affective traits. Using a computational, transdiagnostic approach in two online studies, we found that participants who reported being more affectively reactive across a broad cluster of traits manifested greater frequencies of prosocial actions in two different contexts: They reported being more averse to harming others for profit, and they were more willing to exert effort to benefit others. These findings help illuminate the profile of prosociality across psychiatric conditions as well as the architecture of prosocial behavior in healthy individuals.

Prosocial behaviors are acts that benefit others and often involve some personal cost, such as losing money, suffering pain, or expending effort. These behaviors lie at the core of healthy social relationships and are a key facilitator of social cohesion and group bonding (de Waal, 2008; Fehr & Camerer, 2007; Fehr & Fischbacher, 2003). Nevertheless, the frequencies of these behaviors and the extent to which they are manifested can vary from person to person and in particular seem to be disturbed across a variety of psychiatric disorders (Bartz & Hollander, 2006; Bora, Yucel, & Allen, 2009; Gilbert, 2015; Robson, Repetto, Gountouna, & Nicodemus, 2019). A hallmark of several psychiatric disorders is impairment in affective processing and interpersonal functioning. For instance, criteria for diagnosing depressive, anxiety, and personality disorders (PDs) include affective disturbances such as sadness/irritability, anxiety/fear, and impairment in a range of emotional responses that have a significant impact on social functioning (American Psychiatric Association, 2013). Thus, changes in prosocial behaviors may be one manifestation of a general impairment in interpersonal functioning in these disorders. In fact, previous studies have associated depression, anxiety, and PDs with poor capability in creating and maintaining social relationships, reduced cooperation and reciprocity, and violence toward others and oneself (Blair, 2007; Glenn, Raine, & Schug, 2009; Howard, 2015; King-Casas et al., 2008; Koenigs, Kruepke, & Newman, 2010; Mier et al., 2013; Rilling et al., 2007; Robson et al., 2019; Unoka, Seres, Aspán, Bódi, & Kéri, 2009; Yu, Geddes, & Fazel, 2012). Therefore, affective disturbances in psychiatric disorders negatively impact patients’ social lives, with low prosocial disposition putatively being one of those manifestations.

At the same time, broad affective traits that are not associated with specific psychiatric categories also have been related to prosocial behavior in healthy individuals. For example, people who are less empathetic toward others are less inclined to behave prosocially in a variety of different scenarios (Batson, 2010; Batson, Eklund, Chermok, Hoyt, & Ortiz, 2007; Decety, Bartal, Uzefovsky, & Knafo-Noam, 2016; FeldmanHall, Dalgleish, Evans, & Mobbs, 2015; Hein, Silani, Preuschoff, Batson, & Singer, 2010; Lockwood, 2016; Lockwood, Apps, Valton, Viding, & Roiser, 2016; Lockwood, Seara-Cardoso, & Viding, 2014; Morelli, Rameson, & Lieberman, 2014). In addition, apathy and alexithymia have been associated with reduced motivation to help others (FeldmanHall, Dalgleish, & Mobbs, 2013; Lockwood, Bird, Bridge, & Viding, 2013; Lockwood, Hamonet, et al., 2017; Patil & Silani, 2014a, 2014b). Importantly, even though these traits have been linked to prosocial behavior in healthy participants, they are prevalent across multiple psychiatric disorders. In particular, trait-level apathy and alexithymia have been linked to depression, anxiety, and PDs among other psychiatric conditions (Ang et al., 2018; Chow, 2000; De Berardis et al., 2017; Hendryx, Haviland, & Shaw, 1991; Husain & Roiser, 2018; Lander, Lutz-Zois, Rye, & Goodnight, 2012; Le Heron, Apps, & Husain, 2018; Le Heron, Holroyd, Salamone, & Husain, 2019; Marin, 1990; New et al., 2012; Starkstein & Leentjens, 2008; Strauss & Cohen, 2017; Taylor & Bagby, 2004; Thobois, Prange, Sgambato-Faure, Tremblay, & Broussolle, 2017; van Reekum, Stuss, & Ostrander, 2005), while reduced empathy has been associated with PDs, apathy, and alexithymia (American Psychiatric Association, 2013; Bird & Viding, 2014; Herpertz & Bertsch, 2014; Lander et al., 2012; Lockwood, Ang, Husain, & Crockett, 2017; Lockwood et al., 2013; New et al., 2012; Valdespino, Antezana, Ghane, & Richey, 2017). Therefore, these broader, affective traits may not only modulate social behavior in healthy participants but also play a role in the social dysfunctions and affective impairments across multiple psychiatric disorders and symptoms.

This situation relates to the more general problem of diagnostic categorization in psychiatry, where normal is separated arbitrarily from abnormal, without considering disorders as extreme cases of a mental health continuum (Braun, 2018; McGorry & Nelson, 2016; Newton-Howes, Clark, & Chanen, 2015). To address this challenge, the research domain criteria initiative was developed to identify biologically plausible, transdiagnostic signatures of different psychiatric disorders (Insel et al., 2010). Although aspects of social functioning are included in research domain criteria, prosocial behavior has largely been overlooked, which is surprising given its relevance for healthy social functioning. Therefore, the precise relationship between prosocial deficits and psychopathology has yet to be established—in particular, the specificity of such deficits to distinct psychiatric syndromes versus broader affective traits present across healthy and psychiatric populations. A transdiagnostic approach may help identify specific phenotypes within a dimensional affective and psychiatric domain that underlie deficits in prosociality, addressing the high comorbidity that exists across different disorders and the high multicollinearity between different traits.

Here, we adopted a dimensional approach to investigate the relationship between individual differences in prosocial behavior and both specific psychiatric traits and broader affective traits. We aimed to elucidate, using canonical correlation analysis (CCA), whether specific psychiatric categories can explain prosocial behavior even in a spectrum where broader affective traits are considered. CCA determines the combination of variables (i.e., psychiatric/affective traits) that are predictive of high levels of another combination of variables (e.g., different types of prosocial behavior; Sherry & Henson, 2005). Importantly, CCA can also identify which variables are key predictors, which are collinear with the other variables, and which may be predictive only because they suppress other variables (Nimon, Henson, & Gates, 2010). Here, we used CCA to identify which traits are more strongly linked to prosocial behaviors when multicollinearity and suppression effects are controlled for.

Importantly, most existing studies have measured unidimensional aspects of prosociality, typically related to monetary transactions in economic games or donations to charities (Declerck, Boone, & Emonds, 2013; Koenigs et al., 2010; Rilling, King-Casas, & Sanfey, 2008; Robson et al., 2019). However, the nature of the benefits and costs associated with prosocial actions can vary depending on a given context. For example, people are more willing to assume monetary costs in order to avoid harming others compared to themselves, displaying hyperaltruism (Crockett, Kurth-Nelson, Siegel, Dayan, & Dolan, 2014; Crockett, Siegel, Kurth-Nelson, Dayan, & Dolan, 2017; Crockett et al., 2015). In contrast, people are less willing to incur effort costs to financially benefit others compared to themselves, showing prosocial apathy (Lockwood, Hamonet, et al., 2017). While on the surface, these findings seem opposing, it may be the case that those who are more averse to harming others compared to self are also more willing to put in effort to benefit others. Such common variance would suggest a common, *deep* prosocial disposition underlying both contexts, where moral (hyperaltruism) and motivational (prosocial apathy) aspects of prosocial behavior converge.

To address these questions, we sampled the general population using online measures and tested for shared variance between these two aspects of prosocial behavior and their relationship to affective and psychiatric traits. We adapted for an online setting as used in two previously published lab-based tasks that measured hyperaltruism (harm aversion task; Crockett et al., 2014, 2015, 2017) and prosocial apathy (prosocial effort task; Lockwood, Hamonet, et al., 2017). Using computational modeling of choices in both tasks, we were able to identify participants’ preferences for costly actions that benefited either others or themselves. The aim of Study 1 (*n* = 113) was to examine whether the adapted versions of these tasks could reproduce the same behaviors seen in the lab. In Study 2 (*n* = 212), we aimed to replicate Study 1 within a larger sample. Finally, we collapsed both samples to maximize power to investigate, using CCA, which traits mostly predict prosocial behavior. We focused on specific psychiatric traits, including PD (general, psychopathy, and borderline, the latter two being the subtypes that have been more robustly linked to impaired social cognition and behavior), depression, and anxiety, and broad affective traits, including different dimensions of apathy, alexithymia, and empathy.

## Method

### Participants

Data were collected online using Amazon’s Mechanical Turk (MTurk), and all procedures were administered through Qualtrics.com. Participants received a base-rate payment of $7.85, plus a bonus based on their performance on the prosocial effort task (*M* = $0.90, *SD* = $0.13). All subjects were from the United States and were over 18 years old. Exclusion criteria are detailed below.

For Study 1, we used a sample size based on previous studies that have revealed hyperaltruism and prosocial apathy effects (Crockett et al., 2014, 2015, 2017; Lockwood, Hamonet, et al., 2017), aiming to replicate these lab-based effects in adapted versions of the tasks collected in online samples. We further increased the sample sizes to accommodate the potential noise introduced by adapting these lab-based tasks to online platforms. Thus, in Study 1, 113 participants successfully completed the whole protocol (50 women, age *M* = 35.7, *SD* = 11.6).

For Study 2, we aimed (a) to double the sample size of Study 1 in order to replicate its results and have more statistical power to correlate behaviors in the two tasks and (b) to have enough power to perform the CCA once both studies’ samples were collapsed, in line with recommended sample sizes (Hair, Black, Babin, & Anderson, 2010; Leach & Henson, 2014). Thus, in Study 2, 212 participants successfully completed the whole protocol (101 women, age *M* = 37.7, *SD* = 10.6). When both studies were collapsed, we obtained a total of 325 participants to be included in the CCA.

Participants provided their consent online. Participants’ identities were unknown. The study was approved by the Medical Sciences Interdivisional Research Ethics Committee, University of Oxford (MSD-IDREC-C1-2015–098).

### Procedure

Participants were taken to a link on the Qualtrics website that contained versions of both tasks and self-report measures. Participants performed the self-report measures first, followed by either the prosocial effort task or the harm aversion task. For Study 1, participants always completed the tasks in the same order: harm aversion task first and prosocial effort task second. For Study 2, the orders were counterbalanced: some participants started with the harm aversion task (*n* = 111), while others completed the prosocial effort task first (*n* = 101). Within the self-report section, the different scales were administered in a fully randomized order. Each task followed the same sequence: detailed instructions about the task, followed by practice trials and comprehension questions (see below), and then the main blocks of the experiment.

### Exclusion Criteria and Quality Assurance

First, we identified potential MTurk bots and survey-farmers assigning a score from 0 to 8 to each participant based on their location and qualitative responses, where higher scores mean more likelihood of poor data quality (Prims & Motyl, 2018). Qualitative responses of participants with a score higher than 0 were closely examined to check quality. All participants in our study showed low scores (i.e., ≤ 3) and their responses did not suggest presence of bots or survey-farmers.

To ensure the quality of data collected from the online platform, we followed recommendations from previous studies using Amazon’s MTurk and established exclusion criteria to improve the quality of the data. As such, participants had to successfully answer comprehension questions about the instructions, the attention-check trials, and a cutoff for missed trials (Crump, McDonnell, & Gureckis, 2013). Thus, after a practice set, participants had to answer three comprehension questions about the instructions of the tasks. If the participant answered any of these questions incorrectly, they received an additional summary of the instructions. Then, they were required to answer the same comprehension questions; if any were failed again, the study was terminated. We also applied additional exclusion criteria based on task performance. For the harm aversion task, four attention-check trials were included. In these trials, one of the two options was obviously more attractive compared to the other (higher reward in exchange of less amount of electric shocks). If any of these four attention-check trials was responded to incorrectly, participants were excluded from the final analyses (Study 1, *n* = 2; Study 2, *n* = 19). For the prosocial effort task, participants were excluded if they missed (i.e., decision not made within 6-s time window) more than 10% of the trials (Study 1, *n* = 4; Study 2, *n* = 10). Both tasks had different exclusion criteria due to their characteristics. Thus, we did not include attention-check trials in the prosocial effort task as each decision in this task was followed by a direct action (e.g., work or rest) associated to real outcomes (money). On the other hand, we did not include missed-trial criteria in the harm aversion task because, unlike the prosocial effort task, responses on the task were not time restricted. Finally, participants were only allowed to participate if they were using a laptop with either mouse or trackpad or a desktop computer.

### Behavioral Tasks

We created an online version of a harm aversion task, where participants had to hypothetically imagine that money was being traded off against painful shocks in a laboratory setting, but neither the money nor shocks were actually delivered. For the prosocial effort task, we created a version in which effort was operationalized as ticking boxes on the screen in a fixed time window. The social aspects in both tasks, as well as the bonuses that participants could earn in the prosocial effort task, were fully transparent.

#### Hypothetical harm aversion task

Participants were instructed to imagine a hypothetical experiment in which they were randomly assigned to the role of “decider” and another unknown person was assigned the role of “receiver.” As the decider, they would trade off money against electric shocks that could be delivered either to themselves (self condition) or to the receiver (other condition). Money always was received by the decider. Participants were told to imagine that the electric shocks were comparable to a painful shot at the doctor’s office.

**Figure 1 fig1:**
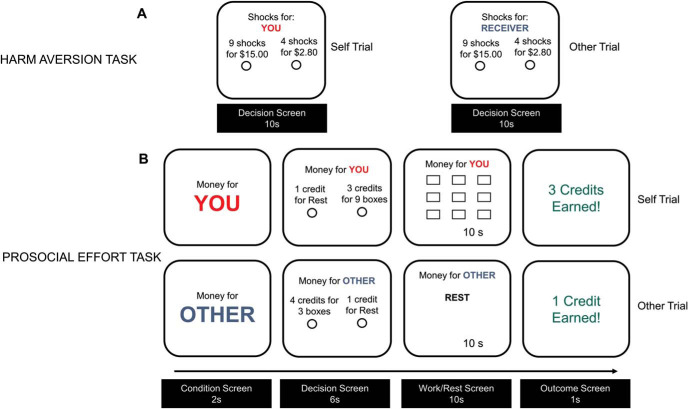
Behavioral online tasks testing two aspects of prosocial behavior. Panel A: Samples of self (left panel) and other (right panel) trials in the hypothetical harm aversion task. Participants had to decide between high and low profit in exchange for a high or low number of electric shocks that could be delivered to either themselves or to an unknown person. The task was hypothetical such that no money or electric shocks were actually delivered. Panel B: Samples of self (top panel) and other (low panel) trials of the prosocial effort task. Participants made choices between a baseline rest and low reward option and a work offer of a variable higher effort (30%, 50%, 70%, and 90% of the boxes ticked in a calibration phase) and higher reward (2–4 credits). If the work offer was chosen, participants had to tick in 10 s the specific number of boxes required to obtain the reward on offer (top panel); otherwise, zero credits were delivered. If the baseline was chosen, participants rested for 10 s and received one credit (low panel). On half of the trials, credits were allocated to participants themselves (self trials), and in the other half, they were allocated to the other person (other trials).

On each trial, participants chose between two options: a harmful option of more shocks and more money or a helpful option that resulted in fewer shocks and less money. The recipient of the shock was listed at the top (red for self trials, blue for other trials; Figure 1A). The harmful and helpful options were randomly allocated to the left or the right to discourage habitual responding. Participants were instructed to make their choices by clicking their mouse and to respond within a maximum of 10 s on each trial. After 10 s remaining on the choice trial, participants were prompted to give an answer.[Fig fig1]

To preserve choice independence, and following previous laboratory procedures, participants were told to imagine that one trial would be randomly selected and actually implemented. However, participants were explicitly told that, in reality, nobody would receive any shock and their participation payment would not be affected by their choices.

Participants were randomly assigned to one of three trial sets following the procedures described in detail elsewhere (Crockett et al., 2014). These three different trial sets help to ensure that results are not a product of the choice set. Each trial set was optimized to give the most efficient estimates of potential participants’ harm aversion parameters. Participants completed 35 trials in both the self and other conditions, with 70 trials in total. Four attention-check trials were included (as exclusion criteria and not included in the final analysis; see above), two for self and two for the other conditions.

#### Prosocial effort task

In this task, participants made decisions about their willingness to exert effort to obtain real monetary rewards for themselves or for another unknown person. On each trial, participants were presented with two options: a “work” offer, associated with different levels of effort (30%, 50%, 70%, and 90% of the maximum number of boxes that a participant could tick in 10 s) and rewards (2–4 credits), and a “rest” option related to no effort and only one credit. All trials were the same duration regardless of what choice was made, ensuring that choices were not influenced by temporal discounting (Critchfield & Kollins, 2001). On half of the 48 trials, participants made choices and exerted effort to benefit themselves, while on the other half, they exerted it to benefit another unknown person. Participants were explicitly told that money on “other trials” would be delivered to another participant in one of our studies, that their identity would remain confidential, and that the other participants did not know that they were receiving a bonus earned by another person’s effort.

Each trial started with a screen indicating the condition (red for self and blue for other trials) for 2 s, followed by a decision screen where participants had to make their choice (Figure 1B). Participants had 6 s to make a decision. If the participants chose the work offer, a screen with the number of boxes to tick appeared. To earn the corresponding number of credits, the participants had to tick all boxes within 10 s, from top to bottom, left to right. If all the boxes were not ticked, then zero credits would be earned. If the rest option was chosen, participants saw a screen with the word “Rest” at the top, and they were not required to execute any task for 10 s. A timer starting from 10 s at the bottom of the screen indicated the time on all trials. Once 10 s had passed, a screen indicating the number of credits earned appeared for 1 s.

To incentivize participants to pay attention to the offers in the task and make active decisions on each trial, two measures were undertaken. First, participants had 6 s to make their initial decision to work or rest, otherwise they would receive zero credits. Second, the location of the work and rest offers was presented in a counterbalanced fashion on the left or right of the screen.

Prior to the beginning of the task, the number of tick boxes used for the effort levels was calibrated to each participant’s skill level. In this calibration stage, participants were asked to tick as many boxes as they could within 10 s, up to 25, from top to bottom and from left to right. This task was repeated three times. Participant were told that they would receive higher bonus payments if they ticked a higher number of boxes. The third trial of calibration was used as a subject-specific threshold for the different levels of effort in the decision task.

### Computational Modeling

We next used computational models to precisely quantify the influence of pain on the devaluation of money in the hypothetical harm aversion task and the influence of effort on the devaluation of money in the prosocial effort task. All parameters used in both the hypothetical harm aversion task—that is, κ_self_, κ_other_, β_self_, and β_other_ (Crockett et al., 2014, 2015, 2017)—and the prosocial effort task—that is, λ_self_, λ_other_, and β (Lockwood, Hamonet, et al., 2017)—were estimated individually for each participant using nonlinear optimization implemented in Matlab (MathWorks) for maximum likelihood estimation.

#### Hypothetical harm aversion task

For the hypothetical harm aversion task, choices were analyzed using a model based on previous studies (Crockett et al., 2014, 2015, 2017) that explains choices by differences in subjective values between harmful and helpful options. The model describes this difference as follows:

ΔV=(1–κ)Δm–κΔs

$$\text{κ}=[\begin{array}{l} {\text{κ}}_{\text{self}}\;\text{if Self trial}\\ {\text{κ}}_{\text{other}}\;\;\text{if Other trial} \end{array}$$

where ΔV is the difference between the subjective value of the harmful and helpful option, and Δm and Δs are objective differences in money and shocks between harmful and helpful options, respectively. In this model, Δm and Δs are weighted by a free parameter κ, which represents harm aversion. When κ is 0, any profit will be accepted regardless of the amount of shocks associated with it. When κ approaches 1, participants become harm averse, sacrificing an increasing amount of profit to avoid additional shocks. Depending on who is receiving the shocks, κ_other_ captures the subjective cost of harming the receiver (other trials), and κ_self_ explains the cost of harming oneself (self trials). A softmax function was used to transform trial-by-trial differences in value into choice probabilities (Daw, 2011):

$$\text{P}\left(\text{choose alternative}\right)=\frac{1}{1+{\text{e}}^{-\text{β}\Delta \text{V}}}$$

where β is a temperature parameter related to the sensitivity of choices according to ΔV, defining the linearity of the slope. As β approaches 0, the slope becomes more linear, meaning more noise in choice probability. When β approaches 100, the sigmoid approximates a step function, making choice preferences deterministic.

#### Prosocial effort task

Choices in the prosocial effort task were fitted using a model that describes the degree to which rewards are parabolically discounted by effort, based on previous studies (Chong et al., 2017; Hartmann, Hager, Tobler, & Kaiser, 2013; Lockwood, Hamonet, et al., 2017). Thus, devaluation of reward by effort for self and other can be represented by the following formula:

$$\text{SV}=\text{R}–{\text{λE}}^{\text{2}}$$

$$\text{λ}=[\begin{array}{l} {\text{λ}}_{\text{self}\;\;\;}\text{if Self trial}\\ {\text{λ}}_{\text{other}\;\;\;}\text{if Other trial} \end{array}$$

where SV is the subjective value of the work offer with a certain reward, R, and effort, E. The degree by which reward is discounted by effort is weighted by the discount parameter λ and is different for self and other trials. When λ is 0, the participant does not discount reward by effort. However, when λ approaches 0.5, the reward is increasingly discounted by effort. We set λ with a maximum of 0.5 because for values beyond this constraint, there is no change in the predicted choice behavior in the task; that is, all offered values are below the value of resting (i.e., 1 credit for 0 effort). SVs were transformed into choice probability using a softmax function as follows:

$$\text{P}\left(\text{i}\right)=\frac{\text{eβ}.\text{SV}\left(\text{i},\text{t}\right)}{\text{e}(\text{β}.\text{1})+\text{e}(\text{β}.\text{SV}\left(\text{i},\text{t}\right))}$$

where i is the subjective value of the work option. 1-P(i) is used to calculate the probability of choosing to rest.

### Self-Report Scales

#### Standardized Assessment of Personality—Abbreviated Scale (SAPAS)

The SAPAS (Moran et al., 2003) is a short screening test that provides a valid measure of the likely presence of general PD. The SAPAS does not focus on one specific dimension of PD, essentially covering the broad multidimensional domain of PDs. Participants respond to eight dichotomously rated (yes/no) items about the person’s normal personality, scoring 0 if the PD trait is absent and 1 if it is present. A score greater than or equal to 3 is used as a cutoff for PD.

#### McLean Screening Instrument for Borderline Personality Disorder (MSI-BPD)

The MSI-BPD (Zanarini et al., 2003) consists of 10 yes/no items that cover the nine diagnostic criteria for BPD according to the *Diagnostic and Statistical Manual of Mental Disorders* (5th ed.; *DSM–5*; American Psychiatric Association, 2013). A score equal to or over 7 would suggest the diagnosis of BPD.

#### Self-Report Psychopathy Scale—Short Form (SRP-4)

The SRP-4 (Paulhus, Neumann, & Hare, 2009) is a 29-item scale that measures psychopathic traits in noninstitutionalized samples. Participants must agree or disagree with each item on a 5-point Likert scale. Four subscales are included in this scale, representing different dimensions of psychopathy: Interpersonal, Affective, Lifestyle, and Antisocial.

#### Depression and Anxiety subscales (extracted from DASS-21)

The Depression Anxiety Stress Scales-21 (DASS-21) is a short form of the original 42-item self-report measure, the Depression, Anxiety, and Stress Scale (Lovibond & Lovibond, 1995). Here, we included only the first two subscales: Depression, associated to dysphoric mood, and Anxiety, related to physical arousal. Each subscale consists of seven items, where participants rate how frequently and severely each statement has been experienced over the previous week on a 4-point scale.

#### Apathy-Motivation Index (AMI)

The AMI (Ang, Lockwood, Apps, Muhammed, & Husain, 2017) is an 18-item scale that measures apathy-motivation. Participants indicate their level of agreement with each item using a Likert scale from 0 to 4. Higher scores mean higher levels of apathy. The AMI comprises three subscales representing different domains of apathy: Behavioral Activation, Social Motivation, and Emotional Sensitivity.

#### Toronto Alexithymia Scale (TAS-20)

The TAS-20 (Bagby, Parker, & Taylor, 1994) has 20 items that are rated using a 4-point scale. This scale is divided into three subscales: Difficulty Describing Feelings, Difficulty Identifying Feelings, and Externally Oriented Thinking.

#### Interpersonal Reactivity Index (IRI)

The IRI (Davis, 1980) is a 28-item scale that measures empathy multidimensionally. Participants respond to each item using a 5-point Likert scale. The IRI comprises four subscales: Perspective Taking, Fantasy, Personal Distress, and Empathic Concern.

### Statistical Analysis of Behavioral Data

Nonparametric Wilcoxon signed-ranks tests were used to test differences in self and other parameters in both tasks as these measures were not normally distributed. Likewise, Spearman’s rank correlation coefficient was used to correlate parameters across tasks. To test significant differences between correlation coefficients, Fisher *Z*-transformation of the absolute values of correlation coefficients was used. For the prosocial effort task, two different analyses of variance were also performed to examine differences between conditions in choice behavior for effort and reward and self versus other trials.

To test the relationship between choice parameters and psychiatric and affective traits, we performed a canonical correlation analysis to examine associations in a transdiagnostic manner across different traits. CCA is a data-driven multivariate approach that finds the maximal correlation between two sets of variables (Sherry & Henson, 2005), generating statistically independent pairs of synthetic variables referred to as canonical variates (CVs). Thus, CCA is a form of data dimensionality reduction that has similarities to factor analysis and principal components analysis. However, the aim of CCA is to find what combination of variables (i.e., a CV) maximally correlates with another combination of variables (i.e., another CV), while factor analysis groups similar variables into dimensions (i.e., factors) with the aim of reducing data complexity.

In the current study, we aimed to identify a CV comprised of affective and psychiatric traits that was maximally correlated with a CV comprised of our prosocial behavior measures. We included SAPAS, MSI-BPD, DASS-21’s Depression and Anxiety scores, as well as the subscales scores of TAS-20, AMI, SRP-4, and IRI for the affective and psychiatric trait CV. For the prosocial domain CV, we included hyperaltruism (κ_other_ − κ_self_) and prosocial effort (reverse-coded prosocial apathy, λ_other_ – λ_self_) parameters in the computational models that quantified degrees of prosociality. The overlap from both sets of variables combines to create a canonical correlation coefficient (*r*) indexing the size of the relationship between the two CVs. By simultaneously considering all the variables in a single analysis, CCA allows us to understand how each trait affects prosocial behavior while avoiding the inflation of experiment-wise Type I error rates that typically occur when multiple univariate analyses are conducted (Sherry & Henson, 2005).

Using CCA, it is possible to determine which variables contribute to explaining a CV, which variables are collinear within a CV, and which may have suppressor effects. Such an approach therefore has significant benefits over simply correlating a range of traits to individual task behaviors. To interpret a CCA, three sets of values are important (Sherry & Henson, 2005). First are the canonical loadings (structure coefficients) of each set of variables onto their own CV. These coefficients are analogous to the structure coefficients used in a factor analysis structure matrix. Second are the canonical cross-loadings, which are the linear correlations between an observed variable from one set and the other CV. Cross-loadings are a more direct measure of how a variable (e.g., an affective or psychiatric trait) affects the predicted dimension (e.g., prosocial behavior in both tasks). Third are the canonical weights, the standardized canonical function coefficients that are used in each linear equation to combine the observed variables for each set into two corresponding CVs, maximizing the correlation between the two CVs. These coefficients are analogous to beta weights in multiple regression. Importantly, discrepancies between these indicators are informative of multicollinearity and suppressor effects (Capraro & Capraro, 2001; Kuylen & Verhallen, 1981; Nimon et al., 2010). Thus, variables with high loadings but low weights may be indicative of multicollinearity–that is, variance in these variables has been explained by other variables in the function coefficients. On the other hand, low loadings and high weights may mean that these variables are suppressing irrelevant variance—that is, variance shared with other variables in their own CV but not with the other CV.

We complemented CCA results with a commonality analysis (Nimon et al., 2010) with those variables that show either high loadings, high weights, or both. This analysis provides the partition of the variance in two different forms: the explanatory power of the unique contribution of each variable and the explanatory ability of that variable that is common with the other variables. In doing so, it is possible to support identification of multicollinearity and suppressors. Prior to running the CCA, questionnaire scores and parameter estimates were *z*-scored across participants. We considered moderate to high loadings (*x* ≥ 0.3 and *x* ≤ −0.3) for interpretation *(*Lambert & Durand, 1975).

All tests were implemented in SPSS24, except Fisher *Z*-transformation, which was performed using the online tool at http://vassarstats.net/rdiff.html. Furthermore, we additionally performed a permutation analysis for statistical significance of the CCA using the p.perm function implemented in the CCP package in R (Roy’s largest root test, 1,000 permutations). Two-tailed tests were used for all analyses.

Finally, the same analyses mentioned above were performed using proportion of choices in self and other trials rather than computational parameters for self and other. Similar results were found with slight differences. These results are reported in the online supplementary materials.

## Results

### Study 1: Measuring Two Forms of Prosocial Behavior

#### Hypothetical harm aversion task: Participants are more averse to harming others for profit compared to themselves

We first tested whether hyperaltruism—greater aversion to harming others for money than oneself—was present in our online hypothetical task. Participants were instructed to imagine that money was being traded off against painful shocks in a laboratory setting. Electric shocks could be hypothetically delivered either to themselves (self condition) or to an unknown person (the receiver; other condition), while money was hypothetically always received by participants. Participants chose between two options: a harmful option of more shocks and more money or a helpful option that resulted in fewer shocks and less money (Figure 1A). Results revealed that participants chose the helpful option significantly more in other (*M* = 46% of trials, *SEM* = 3%) compared to self (*M* = 37%, *SEM* = 2%) trials (*z* = −2.82, *p* < .01; Supplementary Figure S1A), replicating the previously observed hyperaltruism effect.

#### Prosocial effort task: Participants are less willing to choose highly effortful acts that benefit others compared to themselves

The prosocial effort task involved making decisions about the willingness to exert effort to obtain real monetary rewards for oneself or for another person. Here, effort was operationalized as the number of boxes on a screen that could be clicked in a specified order within a time limit (Figure 1B). Crucially, on half of the trials, participants made choices and exerted effort to benefit themselves (self trials), while on the other half, they exerted it to benefit another unknown person (other trials).

Using analyses of variance with proportion of choosing the work option as a dependent variable, we tested whether choosing effortful actions, over rest, to get profit depends on the beneficiary of the reward (self vs. other). We found an Effort × Beneficiary interaction, *F*(2.1, 236.9) = 7.74, *p* < .001, η_p_^2^ = 0.07 (Supplementary Figure S2A), and main effects of effort, *F*(1.7, 185) = 91.43, *p* = < 0.001, η_p_^2^ = 0.45, and beneficiary, *F*(1, 112) = 93.55, *p* < .001, η_p_^2^ = 0.46. Participants were less willing to choose the work option when the levels of effort increased. This effect was amplified when the other person was the beneficiary, with participants less willing to exert effortful actions when the reward was received by the other person compared to themselves. We also found main effects of reward, *F*(1.5, 163) = 48.77, *p* < .001, η_p_^2^ = 0.3, and beneficiary, *F*(1, 112) = 93.55, *p* < .001, η_p_^2^ = 0.46, but no Reward × Beneficiary interaction, *F*(1.7, 189.3) = 3.03, *p* = .59 (Supplementary Figure S2B). Even though participants were less willing to choose to work to benefit others than themselves, the difference between self and other was consistent across the reward levels.

We then used computational models to quantify how rewards were devaluated by pain in the hypothetical harm aversion task and effort in the prosocial effort task. In line with previous studies, we found greater harm aversion for others (*M* = 0.45, *SEM* = 0.03) than self (*M* = 0.37, *SEM* = 0.02; κ_other_ > κ_self_, *z* = −2.28, *p* < .03; Supplementary Figure S1B), suggesting that even when the pain and money are hypothetical, participants give up more money to prevent harm to others compared to self. In the prosocial effort task, participants showed higher devaluation of others’ reward by effort (*M* = 0.11, *SEM* = 0.02) compared to self (*M* = 0.02, *SEM* = 0.01; λ_other_ > λ_self_, *z* = −8.34, *p* < .001; Supplementary Figure S2C), indicating that participants chose to work less to benefit others compared to self. Thus, using computational models of prosocial behavior, we could support the hyperaltruism effect in the harm aversion task, as well as the prosocial apathy effect in the prosocial effort task.

### Study 2: Replicating Hyperaltruism and Prosocial Apathy Effects

In Study 2, we sought to replicate the effects found in Study 1. Thus, in the harm aversion task, participants chose the helpful option significantly more for the other person (*M* = 42% of trials, *SEM* = 2%) compared to self (*M* = 37% of trials, *SEM* = 2%) trials (*z* = −3.0, *p* < .005; Supplementary Figure S1C), replicating Study 1. The model parameters showed effects in the same direction such that participants showed higher aversion to harming others (κ_other_ = 0.41, *SEM* = 0.02) compared to themselves (κ_self_ = 0.36, *SEM* = 0.02), although this trend was not significant (*z* = −1.63, *p* = .10; Supplementary Figure S1D).

For the prosocial effort task, we replicated all effects from Study 1. There were main effects of effort, *F*(1.7, 363.1) = 163.03, *p* < .001, η_p_^2^ = 0.44, and beneficiary, *F*(1, 211) = 187.18, *p* < .001, η_p_^2^ = 0.47, and more importantly, an interaction between effort and beneficiary, *F*(2.3, 475.75) = 20.67, *p* < .001, η_p_^2^ = 0.09 (Supplementary Figure S2D), on the proportion of work choices. Again, we found main effects of beneficiary, *F*(1, 211) = 187.2, *p* < .001, η_p_^2^ = 0.47, and reward, *F*(1.51, 317.9) = 111.3, *p* < .001, η_p_^2^ = 0.35, but no interaction between these variables, *F*(1.9, 398.41) = 1.3, *p* = .27 (Supplementary Figure S2E). Finally, we also found greater discounting for other (*M* = 0.1, *SEM* = 0.01) compared to self (*M* = 0.02, *SEM* = 0.003; λ_other_ > λ_self_, *z* = −11.9, *p* < .001; Supplementary Figure S2F). Importantly, neither hyperaltruism nor prosocial apathy effects were influenced by the order in which the harm aversion and the prosocial effort tasks were completed in Study 2 (see details in the online supplementary materials), suggesting that these effects were robust across task orders and across our two studies.

### Prosocial Behaviors in Both Tasks Are Correlated

**Figure 2 fig2:**
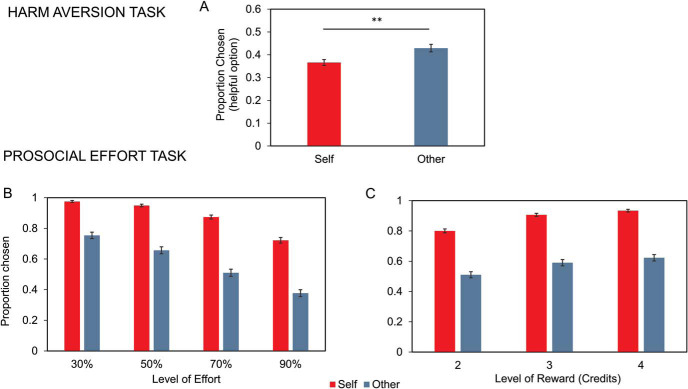
Behavioral results of collapsed samples of Studies 1 and 2 (*n* = 325). Hyperaltruism and prosocial apathy are manifested in the hypothetical harm aversion and the prosocial effort tasks, respectively. Panel A: Proportion of helpful option chosen over the harmful option in the hypothetical harm aversion task. Participants chose in a higher frequency the helpful option for others than themselves. Panel B: Proportion of higher effort-reward work option chosen over the baseline option (rest-lower reward) plotted against effort in the prosocial effort task. Participants chose in a higher frequency the work option for self than others. This difference increased while the effort levels augmented. Panel C: Proportion of higher effort-reward work option chosen over the baseline option (rest-lower reward) plotted against reward in the prosocial effort task. Participants chose in a higher frequency the work option for self than other. Furthermore, they chose more often higher reward options, with an interaction between the target of the reward and the number of credits associated to the option. Error bars depict standard error of the mean. ** *p* < .01.

**Figure 3 fig3:**
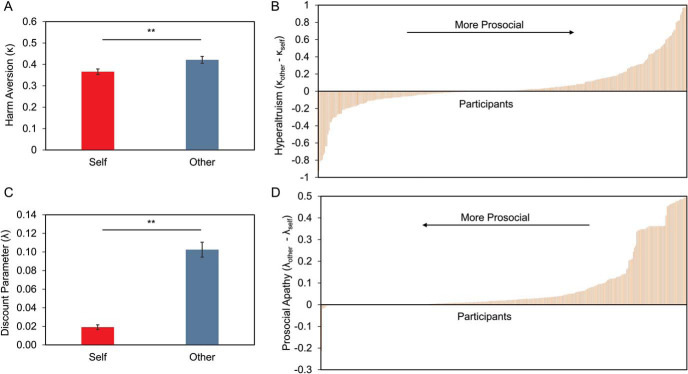
Behavioral-computational results of collapsed samples of Studies 1 and 2 (*n* = 325). Hyperaltruism and prosocial apathy in the hypothetical harm aversion task and prosocial effort task, respectively, are supported by model results. Panel A: Participants were more averse to harm others compared to themselves in the hypothetical harm aversion task (i.e., κ_other_ > κ_self_). Panel B: Distribution of hyperaltruism (i.e., κ_other_ − κ_self_) among deciders in the hypothetical harm aversion task. Panel C: Participants discounted reward to a higher degree by effort when the receiver was the beneficiary compared to themselves in the prosocial effort task (i.e., λ_other_ > λ_self_,). Panel D: Distribution of prosocial apathy (i.e., λ_other_ − λ_self_) among deciders in the prosocial effort task. Error bars depict standard error of the mean. ** *p* < .01.

For correlation analyses, we collapsed both samples in order to maximize statistical power. To begin, we examined the hyperaltruism and prosocial apathy effects in the combined sample (*n* = 325). We found consistent results with those revealed by taking the samples separately in both proportion of choices (Figure 2A for harm aversion task; Figure 2B and 2C for prosocial effort task) and computational parameters (Figure 3A and 3B for harm aversion task; Figure 3C and 3D for prosocial effort task; see online supplementary materials for details).[Fig fig2][Fig fig3]

**Figure 4 fig4:**
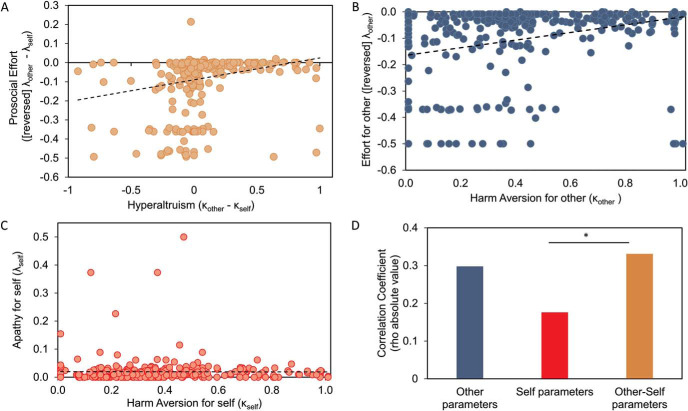
People who are more averse to harm others are more willing to initiate effortful actions to help others. Panel A: Significant correlation between hyperaltruism and prosocial effort (ρ = 0.33, *p* < .001), suggesting deep prosocial disposition across tasks. Panel B: Significant correlation between reversed λ_other_ parameter in the prosocial effort task and κ_other_ in the hypothetical harm aversion task (ρ = 0.3, *p* < .001). Panel C: Significant correlation between λ_self_ and κ_self_ (ρ = 0.18, *p* < .01). Panel D: Absolute values of the correlation coefficients depicted in A, B, and C. Correlation of self parameters was significantly different from correlation between prosocial effort and hyperaltruism. * *p* < .05.

Is there a relationship between prosocial behavior across both tasks? For the correlation analyses, we reverse-coded the prosocial apathy effect (λ_other_ − λ_self_) in the prosocial effort task, and we called this variable *prosocial effort*. We also reverse-coded λ_other_. Thus, higher values were associated with more prosocial decisions in both tasks. Hyperaltruism (κ_other_ − κ_self_) and prosocial effort were positively correlated (ρ = 0.33, *p* < .001; Figure 4A), suggesting that participants who were prosocial in one task were also prosocial in the other. Moreover, we also found a positive correlation between κ_other_ and λ_other_ parameters, showing that participants who were more averse to harming others were more willing to exert effort to benefit others (ρ = 0.3, *p* < .001; Figure 4B). However, we also found a correlation between κ_self_ and λ_self_, although weaker than those revealed in social contexts (ρ = 0.18, *p* < .01; Figure 4C). This result suggests that participants who hypothetically accepted more shocks to gain profit (i.e., less averse to harm themselves) were more willing to work to obtain reward (i.e., discounting in a lower degree reward by effort). We then tested if this correlation of “self” parameters was significantly weaker than the correlation between prosocial manifestations in both tasks. We found that the correlation between self parameters was significantly different from the correlation between hyperaltruism and prosocial effort (*z* = 2.11, *p* < .05; Figure 4D). No significant difference was found between the correlation of self parameters and the correlation of “other” parameters (*z* = 1.64, *p* = .1; Figure 4D). These results suggest that there is a *deep* prosocial disposition common across tasks—people who avoid (hypothetically) harming others also more readily exert effort to earn rewards for others—and this cannot be explained only by effort and pain sensitivity for oneself being linked. Importantly, correlations between prosocial behavior and other parameters across both tasks are consistently found if the sample is split in Studies 1 and 2, suggesting that this relationship is robust (see online supplementary materials for details).[Fig fig4]

### Transdiagnostic Relationship Between Affective and Psychiatric Traits and Prosocial Dispositions

To examine the traits associated with higher prosocial behavior across contexts, we performed a CCA between participants’ scores in the affective and psychiatric scales and pro-social behavior in both tasks. We aimed to identify what constellation of affective and psychiatric traits are more associated with a CV comprised of our prosocial behavior measures (see “Method”). For the psychiatric and affective traits CV, we included general PD, borderline PD, depression and anxiety trait scores, as well as multiple dimensions of apathy, alexithymia, psychopathy, and empathy traits. Descriptive statistics of each trait in our collapsed sample can be found in Supplementary Table S1, including means and dispersion information. Importantly, all variables show considerable variance across the whole scoring range. On the other hand, for the prosocial CV, we included hyperaltruism (κ_other_ − κ_self_) and prosocial effort (reversed λ_other_ – λ_self_) parameters in the computational models that quantified degrees of prosociality.

Two significant canonical correlations were generated in our analysis as, in CCA, the number of canonical correlations is equal to the number of variables in the smaller of the two sets, first canonical correlation: *r* = .37, Wilks’ λ = 0.79, *F*(36, 610) = 2.18, *p* < .001, second canonical correlation: *r* = .3, Wilks’ λ = 0.91, *F*(17, 306) = 1.72, *p* < .05. However, given that the first canonical function explained most of the variance in the canonical solution, and that the second canonical correlation had prosocial behavior for each task loading in opposite directions (i.e., with one loading positively and the other negatively, not extracting the same variance across tasks), we further analyzed and interpreted results related only to the first significant canonical function. To add robustness to our results, we performed a permutation Roy’s largest root test for significance on this first canonical correlation, supporting a significant association between prosocial behaviors and psychiatric and affective traits (1,000 permutations; *p* < .001).

We looked at loadings (structure coefficients), cross-loadings, and weights (function coefficients) to interpret the results revealed by CCA. We further complemented CCA results with a commonality analysis on those variables that show either high loadings, high weights, or both in order to support identification of multicollinearity and suppressor suggested by CCA (see “Method”).

Consistent with simple correlations, we found a shared prosocial variance across tasks, with prosocial effort and hyperaltruism strongly (>0.3) loaded onto their own CV, suggesting that this dimension encapsulated both measures of prosociality—that is, deep prosocial disposition (loadings: hyperaltruism = 0.79; prosocial effort = 0.77). Furthermore, both showed high weights, consistent with the loading results (weights: hyperaltruism = 0.65; prosocial effort = 0.62).

**Figure 5 fig5:**
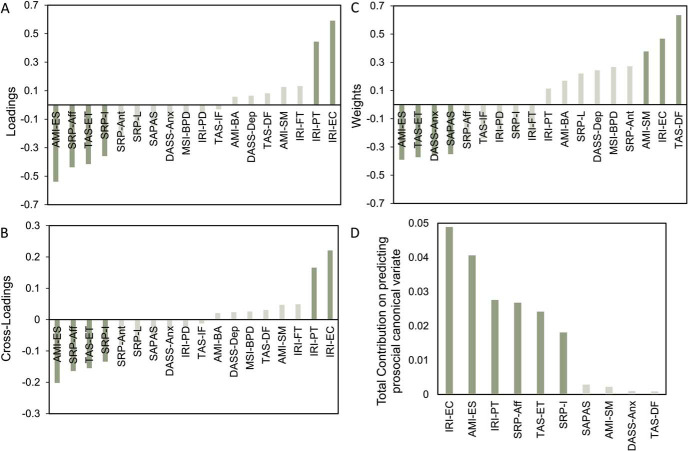
Constellation of traits that are correlated with prosocial behavior. Panel A: Loadings of variables in the affective and psychiatric CV. Panel B: Cross-loadings of variables with the prosocial CV. Panel C: Weights of variables in the affective and psychiatric CV. Note the discrepancies with the loadings suggesting multicollinearity and suppressor effects. Panel D: Total effects of variables with high loadings and weights of the affective and psychiatric traits CV on the prosocial CV. This supports what was found by weights and loadings: Empathic concern and emotional apathy are the most important variables in predicting prosociality. SRP-I = Self-Report Psychopathy Scale Interpersonal; SRP-Aff = SRP Affective; SRP-L = SRP Lifestyle; SRP-Ant = SRP Antisocial; DASS-Dep = Depression Anxiety Stress Scales-21 Depression; DASS-Anx = DASS-21 Anxiety; TAS-DF = Toronto Alexithymia Scale Difficulty in Describing Feelings; TAS-IF = TAS Difficulty in Identifying Feelings; TAS-ET = TAS Externally Oriented Feelings; AMI-ES = Apathy-Motivation Index Emotional Sensitivity; AMI-BA = AMI Behavioral Activation; AMI-SM = AMI Social Motivation; IRI-FT = Interpersonal Reactivity Index Fantasy; IRI-EC = IRI Empathic Concern; IRI-PT = IRI Perspective Taking; IRI-PD = IRI Personal Distress.

Regarding the psychiatric and affective CV, we found a consistent effect of empathic concern being positively, and emotional apathy together with externally oriented thoughts (a dimension of alexithymia) negatively, loaded onto their own CV and cross-loading with the prosocial CV (Figure 5A and 5B). These effects were consistently found looking at the weights and the results revealed by the commonality analysis (Figure 5C and 5D; Table 1). Thus, people with higher levels of empathic concern and lower levels of emotional apathy, together with more attention to their internal feelings, were more averse to harming others and more willing to put in effort to benefit others.[Fig fig5][Table tbl1]

Not all variables showed consistently high values across loadings and weights. Discrepancies between both measures are not uncommon and arise due to multicollinearity between variables and suppression effects across the trait measures. Weights are the contribution of a variable to the canonical solution as a whole; as such, they are sensitive to the presence of multicollinearity. Loadings, on the other hand, are not affected by multicollinearity as they correspond to the unique contribution of each trait to their own affective/psychiatric CV computed separately. Thus, some traits can have small weights because the variance of these variables could have been explained by other traits in the equation (Kuylen & Verhallen, 1981).

Furthermore, high weights do not necessarily mean that there is a high contribution of that trait to the prediction of prosocial behavior. Variables with high weights but low loadings have been suggested as suppressors in linear equations (Capraro & Capraro, 2001; Nimon et al., 2010), meaning that traits showing such a pattern would not be directly related to prosocial behavior, but rather they would be subtracting irrelevant variance of other traits in the affective/psychiatric CV to increase their predictive power in relation to the prosocial behavior CV.

We found that perspective taking contributed positively to its own CV, while interpersonal and affective psychopathy contributed negatively (Figure 5A). However, when examining the weights and the commonality analysis, it is apparent that this is because these variables were collinear with other variables in the CV (Figure 5C and 5D; Table 1). At the same time, although general PD, anxiety, social apathy, and one dimension of alexithymia showed high weights (Figure 5C), they did not contribute highly to their CV (Figure 5A), suggesting suppressor effects, which is supported by their negative common effects and marginal total effects in the commonality analysis results (Figure 5D; Table 1).

In summary, emotional apathy, empathic concern, and externally oriented thinking appear to have a reliable relationship with prosocial behavior, with the former two being predominant such that higher levels of empathic concern and lower degrees of emotional apathy are associated with prosocial behavior across tasks. Furthermore, and collinear with these variables, perspective taking and affective dimensions of psychopathy were also related to prosocial behavior. This suggests there is a broad cluster of affective traits that is associated with prosocial behavior across different contexts.

Importantly, we also performed CCA and commonality analysis using proportion of choices instead of computational parameters as measures of prosocial behavior. Using this approach, we found similar results to those reported in detail here, suggesting that the association between this affective cluster of traits and prosocial behavior in both tasks is robust (see online supplementary materials for details).

## Discussion

Here, we examined the relationship between affective and psychiatric traits and prosocial behavior in two different contexts: aversion to harming others and exerting effort to benefit others. Using a transdiagnostic approach, we found that higher levels of empathic concern and lower levels of emotional apathy were the traits that were most associated with prosocial behavior across contexts. Furthermore, we found that affective aspects of psychopathy and alexithymia were associated with less prosocial behavior, while perspective taking also facilitated prosociality. Notably, no specific psychiatric traits, except for the affective component of psychopathy, were associated with prosocial behavior when controlling for suppression and multicollinearity effects. Taken together, these results suggest that affective reactivity across a broad cluster of traits including apathy, psychopathy, and empathy are related to a deep prosocial disposition.

We showed that high empathic concern and low emotional apathy were the strongest predictors among several affective and psychiatric traits putatively suggested to relate to prosocial behavior. Intriguingly, empathy was a facilitating factor even when traits associated with obstructing prosocial behavior were also included, suggesting that empathic concern is a powerful variable to trigger prosocial behavior over and above individual differences in psychiatric disorders and other affective traits. This is in line with dimensional models for PD used in the *DSM–5*, where impairment in empathy is a major trait used to identify pathological disturbances in interpersonal functioning (American Psychiatric Association, 2013). We also found that low levels of emotional apathy predicted more prosocial decisions. Thus, this would suggest that motivation to engage emotionally, alongside empathic feelings, contributes to prosocial behavior (Decety & Cowell, 2014a, 2014b). In order to recognize and care about others’ emotions and act accordingly, people may have to be sensitive to experience emotions and be motivated by them. In this way, emotional apathy and empathy are constructs that may share common processes (Lockwood, Ang, et al., 2017; Zaki, 2014) and together may be necessary to facilitate prosocial behavior.

Other traits were also related to prosocial behavior—namely, perspective taking—and aspects of alexithymia and psychopathy. Perspective taking is positively related to prosocial behavior, similar to empathic concern, consistent with the importance of understanding the emotions of others in order to help them, a more cognitive aspect of empathic processes (Tamnes et al., 2018; Tusche, Böckler, Kanske, Trautwein, & Singer, 2016). However, empathic concern, a more affective aspect of empathy, had a more predominant role in predicting prosocial behavior. In fact, we found that emotional components across different traits were associated with prosocial behavior. Thus, only emotional apathy, previously linked to affective but not cognitive empathy (Lockwood, Ang, et al., 2017), was associated with prosocial behavior in our results. Furthermore, we found that people who do not avoid thinking about their emotions were more likely to be prosocial. In fact, previous evidence has shown that externally oriented thinking is associated with callous traits in psychopathy (Lander et al., 2012), low empathic concern (Grynberg, Luminet, Corneille, Grèzes, & Berthoz, 2010; Guttman & Laporte, 2002), less reaction to affective stimuli (Wiebe, Kersting, & Suslow, 2017), and alterations in moral reasoning and prosocial behavior (Gleichgerrcht, Tomashitis, & Sinay, 2015; Mannarini, Balottin, Toldo, & Gatta, 2016). Finally, the only specific psychiatric trait predicting prosocial behavior in our data was the affective components of psychopathy, associated with callous feelings, but not its behavioral aspects (e.g., antisociality, impulsivity), in line with previous studies (Lockwood, Hamonet, et al., 2017; Vahl et al., 2014; White, 2014). Taken together, our transdiagnostic approach identified a deep prosocial disposition characterized mostly by affective sensitivity—people who engaged affectively with themselves and others were more likely to assume costs to benefit other people.

We did not find, except for the callous component of psychopathy, effects of specific psychiatric traits in prosociality. Psychopathy is not a clear category in classic psychiatric classification, being traditionally associated with antisocial PD that typically focuses on more behavioral aspects of psychopathy rather than its callous symptomatology (Crego & Widiger, 2015). However, new dimensional conceptualizations of PD, also included in the *DSM–5*, that represent extremes along trait-dispositional continua have captured callous traits, among others, to define psychopathic symptomatology (American Psychiatric Association, 2013; Miller, Lamkin, Maples-Keller, Sleep, & Lynam, 2018; Strickland, Drislane, Lucy, Krueger, & Patrick, 2013). Our results support this conceptualization, suggesting that strict clinical categorizations may not be necessary to understand variability in prosocial behavior. Rather, a constellation of broad traits associated with affective sensitivity across psychiatric and healthy populations may better predict prosociality, suggesting that extreme cases of low affective reactivity could derive from social dysfunction. Thus, our results may shed light on identifying risk and protective factors in a wide spectrum of traits for keeping healthy social relationships. Social dysfunction is a transversal symptom across many psychiatric disorders, and our findings could help illuminate a different perspective to understand and approach this problem through the propensity for prosocial behavior.

Our analytical approach also allowed us to address the multicollinearity that exists across different traits, a problem that has been identified in the psychiatric literature. For instance, our findings suggested that some relationships between traits and prosocial behavior in our study—for example, PD and anxiety—are due to shared variance with other traits but not with prosocial behavior itself, consistent with some previous findings (Gleichgerrcht & Young, 2013; Lander et al., 2012; Mannarini et al., 2016; Vahl et al., 2014; White, 2014). This is in line with our claim that broad affective traits may better explain variability in prosocial behavior than categorical psychiatric disorders. Furthermore, we also found that traits such as empathic concern and emotional apathy are collinear with other traits but at the same time show strong associations with prosocial behavior. Therefore, these results open a potential line of research to disentangle the complex relationship between traits and other traits, traits and behavior, and traits and specific psychiatric disorders. Future research may shed light on how the interaction across different traits can influence ultimate prosocial decisions.

Finally, we found that harm aversion for others and prosocial effort were correlated, suggesting some shared decisional processes across contexts. Importantly, these two aspects of prosocial behavior were more strongly correlated than sensitivity to harm and effort for oneself. These findings point to a common mechanism for making prosocial decisions in two distinct contexts. Social neuroscience has shown shared neural mechanisms underpinning prosocial behavior across different scenarios (Crockett, Clark, Hauser, & Robbins, 2010; Declerck et al., 2013; Rilling et al., 2008; Wood, Rilling, Sanfey, Bhagwagar, & Rogers, 2006), making plausible a shared decisional process at some degree. However, how prosocial behavior is manifested in each context may depend on how the brain processes different outcomes with opposite valences such as reward and harm (Palminteri & Pessiglione, 2016) and the neural representation of these outcomes for self and others (Apps & Ramnani, 2014; Lamm, Decety, & Singer, 2011; Lockwood et al., 2016; Mobbs et al., 2009; Morelli, Sacchet, & Zaki, 2015). Deep prosocial dispositions may involve areas related to social cognition and executive control that may regulate and integrate reward and punishment systems in the brain to execute a prosocial action (Lieberman, 2007; Ruff & Fehr, 2014). In our case, prosocial behavior across both contexts may suggest that individual differences in moral norms reflected by hyperaltruism are associated with variation in how people are incentivized by others’ rewards (and then others’ welfare). Therefore, our results suggest that prosocial behavior includes motivational and moral components, which are modulated by affective sensitivity. Nevertheless, it is still an open question as to what extent motivation to improve others’ welfare shapes our own moral principles and how our moral motives guide our behavior toward benefiting others (Batson, 2008; Haidt, 2007). Future research may shed light on the cognitive and neural mechanisms that underlie both components in prosocial behavior.

It is important to note that the general, deep prosocial behavior suggested by the shared variance of decisions in both tasks may not reflect all aspects of prosociality. We chose two different dimensions of prosocial behavior in order to capture a wide spectrum of decisions: a more moral, normative aspect associated with harm aversion (Crockett et al., 2014; Smith, 1790/2002) and a more motivational, goal-directed dimension associated with effort-rewards trade-off (Lockwood, Hamonet, et al., 2017). In everyday life, people make decisions like these very frequently, such as avoiding going to socially rewarding events in order to avoid contagion of other people when one is sick (harm aversion) or helping a colleague solve a work-related problem (prosocial effort). Thus, we believe that the shared variance between both dimensions of prosociality could cover a wide range of potential prosocial manifestations. Nevertheless, correspondence between lab-based tasks and decisions in everyday life has not been extensively researched, and some caution should be taken in terms of their generalizability to other aspects of prosocial behavior. Future research should expand the current results, adding ecological momentary assessments (Hofmann, Wisneski, Brandt, & Skitka, 2014) to complement the approaches used here and to clarify the correspondence between lab-based decisions and real-world altruism.

## Conclusion

In this study, we have shown that people display prosocial behavior in two different contexts that are modulated by individual differences in broad affective traits. People who showed a configuration of traits characterized by high empathy and affective reactivity showed more aversion to harming others and were more willing to exert effort to benefit others. Reduced empathic feelings and affective sensitivity are present in a variety of psychiatric disorders, suggesting that these features may be associated with poor social relationships seen in a high number of mental health conditions. Thus, these findings shed light on the traits that may underlie social dysfunction in general and clinical populations using a computational, transdiagnostic approach, with the potential of aligning clinical categorization with cognitive-based evidence, thereby improving diagnostic accuracy, identifying high-risk populations, and setting more guided systematic treatments that improve people’s social relationships and mental health.

## Supplementary Material

10.1037/emo0000813.supp

## Figures and Tables

**Table 1 tbl1:** Commonality Analysis Results

Scales	Unique	Common	Total
SAPAS	0.0065	−0.0035	0.0029
SRP-I	0.0003	0.0178	0.0181
SRP-Aff	0.0001	0.0267	0.0268
DASS-Anx	0.0025	−0.0016	0.0010
TAS-DF	0.0258	−0.0249	0.0009
TAS-ET	0.0102	0.0140	0.0242
AMI-ES	0.0043	0.0363	0.0406
AMI-SM	0.0112	−0.0090	0.0022
IRI-EC	0.0096	0.0393	0.0489
IRI-PT	0.0010	0.0267	0.0276
*Note*. SAPAS = Standardized Assessment of Personality—Abbreviated Scale; SRP-I = Self-Report Psychopathy Scale Interpersonal; SRP-Aff = SRP Affective; DASS-Anx = Depression, Anxiety, and Stress Scale Anxiety; TAS-DF = Toronto Alexithymia Scale Difficulty in Describing Feelings; TAS-ET = TAS Externally Oriented Feelings; AMI-ES = Apathy-Motivation Index Emotional Sensitivity; AMI-SM = AMI Social Motivation; IRI-EC = Interpersonal Reactivity Index Empathic Concern; IRI-PT = IRI Perspective Taking.
