# Supplementary material for: Prosocial Behavior Is Associated With Transdiagnostic Markers of Affective Sensitivity in Multiple Domains
Source: Emotion. 2020 Jul 27;22(5):820–35. doi: 10.1037/emo0000813 (PMC9301775; doi:10.1037/emo0000813)
Supplement: Supplementary file 1 [file EMO-2019-1690_Supplemental_Materials.docx]

**Supplementary Material**

**
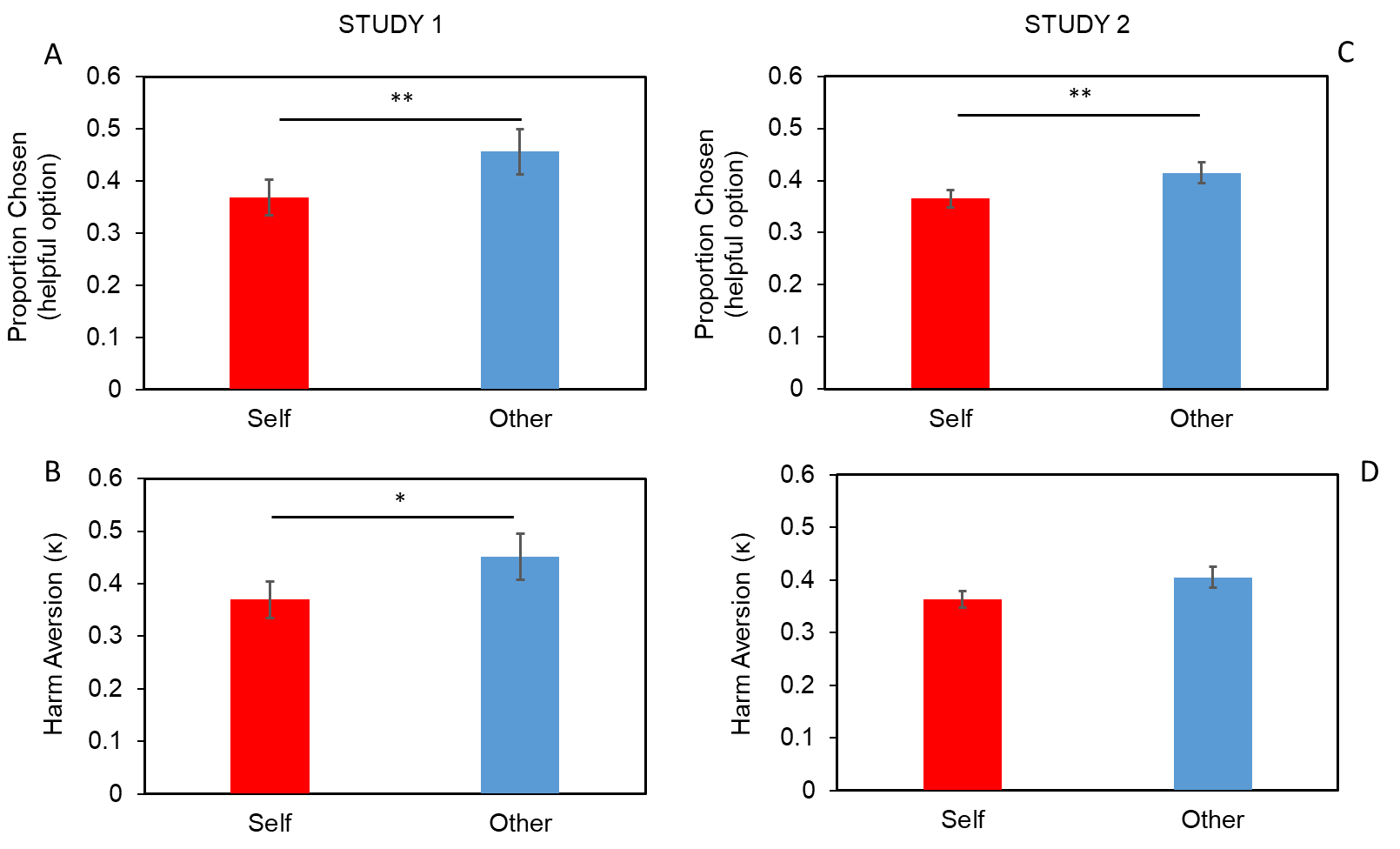
Supplementary Figures**

**Figure S1. Behavioural and computational results in the hypothetical harm aversion task for studies 1 (A and B) and 2 (C and D).** A. Proportion of helpful option chosen over the harmful option in study 1. Participants chose in a higher frequency the helpful option for others than themselves. B. Participants were more averse to harm others compared to themselves in study 1 (i.e. κ_other_ > κ_self_). C. Study 2 replicates behavioural (i.e. proportion of helpful option chosen) results of study 1. D. However, even though participants were more averse to harm others compared to themselves in study 2 (i.e. κ_other_ > κ_self_), this result was not significant using computational parameters. Error bars depict S.E.M. * p < 0.05; ** p < 0.01.

**
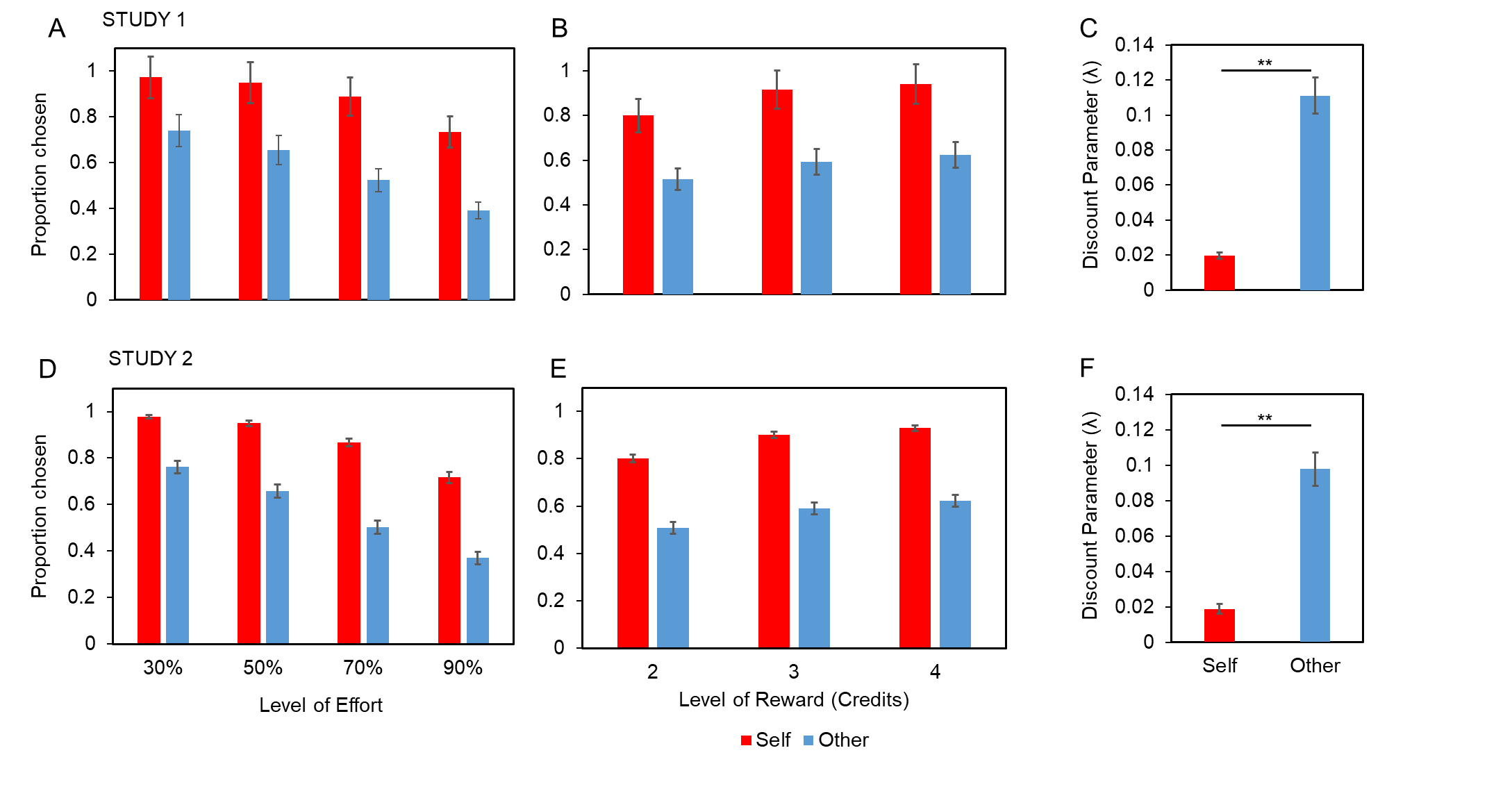
Figure S2. Behavioural and computational results in the prosocial effort task for study 1 (A, B and C) and 2 (D, E and F).** A. Proportion of higher effort-reward ‘work’ option chosen over the baseline option (rest-lower reward) plotted against effort in study 1. Participants chose in a higher frequency the ‘work’ option for self than other. This difference increased while the effort levels augmented. B. Proportion of higher effort-reward ‘work’ option chosen over the baseline option (rest-lower reward) plotted against reward in study 1. Participants chose in a higher frequency the ‘work’ option for self than other. Furthermore, they chose more often higher reward options, but there was no interaction between the target of the reward and the number of credits associated to the option. C. Participants discounted reward to a higher degree by effort when the receiver was the beneficiary compared to themselves in study 1 (i.e. λ_other_ > λ_self_). D, E and F. Study 2 fully replicates behavioural (D and E) and computational (F) results of study 1. Error bars depict S.E.M. ** p < 0.01.

**Supplementary Results**

**1. Task order effects in Study 2:** In order to test whether there was any effect of task order in the harm aversion and prosocial effort tasks, we performed a 2x2 mixed ANOVA having beneficiary/recipient as a within subject factor (self, other) and task order as a between subject factor (harm aversion task first, prosocial effort task first). We performed this analysis on both proportion of choices on one hand, and computational parameters on the other hand for both tasks separately. Proportions of choices and computational parameters revealed similar results. For the prosocial effort task, there were not effects of task order (neither main effect nor interaction, p > 0.1), and only a main effect of beneficiary (Proportions: F_1,210_ = 189.7, p < 0.001, η_p_^2^ = 0.48; Parameters: F_1,210_ = 77.9, p < 0.001, η_p_^2^ = 0.27). However, for the hypothetical harm aversion task we found, together with a main effect of recipient (Proportions: F_1,210_ = 7.6, p < 0.01, η_p_^2^ = 0.04; Parameters: F_1,210_ = 5.1, p < 0.05, η_p_^2^ = 0.02), a main effect of task order (Proportions: F_1,210_ = 15.2, p < 0.01, η_p_^2^ = 0.07; Parameters: F_1,210_ = 14.8, p < 0.01, η_p_^2^ = 0.07), with people being more averse to harm themselves and others when this task was completed first (Proportions: M = 0.45, SEM = 0.2; Parameters: M = 0.44, SEM = 0.2) rather than second (Proportions: M = 0.33, SEM = 0.2; Parameters: M = 0.32, SEM = 0.2). Nevertheless, and crucially, we did not find an interaction between recipient and task order (p > 0.6), suggesting that the hyperaltruism effect was not influenced by the order in which the tasks were completed.

**2. Hyperaltruism and Prosocial Apathy in the combined sample (n = 325):** We found consistent results with those revealed by taking the samples separately. Thus, participants chose in a higher proportion the helpful option in other (M: 43%, SEM: 2%) than self (M: 37%, SEM: 1%) trials (z = -4.09, p < 0.001, Fig.2A). This was supported by a comparison between κ_self_ and κ_other_ showing that participants were significantly more averse to harming other (M: 0.42, SEM: 0.02) compared to themselves (M: 0.37, SEM: 0.01; z = -2.62, p < 0.01, Fig.3A and 3B).

We also replicated the results found in the prosocial effort task. Thus, main effects of beneficiary (F_1, 324_ = 281.27, p < 0.001, η_p_^2^ = 0.47), effort (F_1.7, 552.78_ = 252.97, p < 0.001, η_p_^2^ = 0.44) and their interaction (F_2.2, 714.68_ = 28.13, p < 0.001, η_p_^2^ = 0.08; Fig.2B) were found, as well as main effects of reward (F_1.5, 481.38.2_ = 158.64, p < 0.001, η_p_^2^ = 0.33), beneficiary (F_1, 324_ = 281.27, p < 0.001, η_p_^2^ = 0.47) and their interaction (F_1.84, 596.77_ = 3.73, p < 0.03, η_p_^2^ = 0.01; Fig.2C). Comparing model parameters we also found that participants significantly discounted more reward by effort in other (M: 0.1, SEM: 0.01) than self (M: 0.02, SEM: 0.002) trials (z = -14.51, p < 0.001, Fig.3C and 3D).

**3. Correlation between computational parameters in study 1 and 2:** Hyperaltruism and prosocial effort (κ_other_ - κ_self_ and reverse-coded λ_other_ - λ_self_ for hypothetical harm aversion and prosocial effort tasks respectively) were positively correlated in study 1 (rho = 0.38; p < 0.001) and study 2 (rho = 0.3; p < 0.001), consistent with what was found when both samples were collapsed (see Results in the main text). This is also true for the relationship between κ_other_ and reverse-coded λ_other_ parameters, with positive correlations in study 1 (rho = 0.33; p < 0.001) and study 2 (rho = 0.28; p < 0.001), meaning that participants who showed more aversion to harming others decided more frequently to exert effort to help others. However, we did not find a significant correlation between κ_self_ and λ_self_ in study 1 (rho = 0.14; p = 0.13). Only study 2 revealed a significant relationship between these parameters (rho = 0.19; p < 0.01) although weaker than those found in social contexts.

**4. Model-free analyses:** We performed similar analyses on the collapsed sample using proportion of choices instead of computational parameters in order to support our results with measures that do not consider computational assumptions.

First, we tested whether participants chose in a higher proportion to work for themselves than for others. Indeed, we found that participants were more willing to work to benefit themselves (M: 88%, SEM: 1%) than others (M: 58%, SEM: 2%) when we collapsed choices across effort and reward (z = -13.477; p < 0.001; n= 325).

Next, we tested whether the proportion of helping in the harm aversion task was correlated with choosing to work in the prosocial effort task for self, other and the difference between them (i.e. ‘prosocial preference’, difference between the proportion of choosing to help/work in the other condition and the proportion of choosing to help/work in the self condition). We found that prosocial preferences were positively correlated in both tasks (rho = 0.35; p < 0.001). Likewise, participants who were averse to harm others were more willing to work to benefit others (rho = 0.33; p < 0.001). Finally, people who were less willing to sacrifice money to avoid pain were more laborious to gain profit, suggesting sensitivity to reward across tasks (rho =- 0.13, p < 0.05). Importantly, correlations between choices in other trials (z = 2.7; p < 0.01) and prosocial preferences (z = 3.0; p < 0.005) in both tasks were significantly different from the correlation of choices in the self trials across tasks. However, no significant difference was found between the correlation of prosocial preferences and the correlation of choices in other trials in both tasks (z = -3.0, p = 0.8). These results support computational findings, suggesting that prosocial choices are correlated in both tasks, and this relationship is not associated to reward sensitivity.

Finally, we conducted a CCA to identify the psychiatric and affective traits that modulate prosocial preferences across contexts. We examined the relationship between affective and psychiatric traits, where we included the same scales and subscales described in the main text, and prosocial behaviour, where we included prosocial preferences in both tasks instead of computational parameters. The CCA revealed two canonical correlations, with both reaching significance (first, r = 0.42, Wilks’s lambda = 0.76, F_36, 610_ = 2.53**;** p < 0.001; second, r = 0.29, Wilks’s lambda = 0.91, F_17, 306_ = 1.7; p < 0.05). Given that most of the variance was explained by the first canonical solution, and that prosocial preferences were loading in opposite directions in the second canonical correlation, we just focused on the first canonical correlation for further analyses.

Prosocial preferences in both tasks were highly loaded in the prosocial CV (prosocial effort = 0.91, harm aversion = 0.65), and they congruently showed high weights (prosocial effort = 0.8, harm aversion = 0.42). However, in the affective and psychiatric trait CV there were important differences in the contribution of each variable. Table S2 summaries these results. Emotional apathy, affective psychopathy and alexithymia (externally oriented feelings) were negatively loading in their CV, while empathic concern and perspective taking were contributing positively. Cross-loadings supported these results, with emotional apathy and empathic concern as the strongest variables contributing to predict prosocial preferences (in opposite directions). However, there were some incongruences between loadings and weights. While emotional apathy, affective psychopathy and externally oriented feelings had consistently high contributions across structure and function coefficients, other variables that had low loadings showed high weights, namely SAPAS, antisocial psychopathy, anxiety, difficulties in describing feelings, social apathy, and personal distress, while others showed the opposite pattern, i.e. empathic concern and perspective taking.

These incongruences suggest suppression effects and multicollinearity. In order to support this claim, a commonality analysis was performed with those variables that had high loadings, weights or both. Table S3 summarises these results, which support this interpretation. Finally, emotional apathy and empathic concern were the variables with highest total effects, suggesting that they are the traits that contribute the most to predict prosocial preferences (in opposite directions), supporting the results found using computational parameters.

**Supplementary Tables**

**Table S1. Descriptive information of psychiatric and affective traits included in the canonical correlation analysis.**

|  | Mean | SD | Range | Scoring Range |
| --- | --- | --- | --- | --- |
| SAPAS | 2.8 | 1.9 | 8 | 0-8 |
| MSI-BPD | 2 | 2.6 | 10 | 0-10 |
| SRP-I | 12.9 | 5.4 | 27 | 1-35 |
| SRP-Aff | 13.4 | 5 | 21 | 1-35 |
| SRP-L | 13 | 5 | 21 | 1-35 |
| SRP-Ant | 9.7 | 3.8 | 21 | 1-35 |
| DASS-Dep | 3.9 | 5.2 | 21 | 0-21 |
| DASS-Anx | 2.4 | 3.5 | 19 | 0-21 |
| TAS-DF | 11.3 | 4.4 | 20 | 1-25 |
| TAS-IF | 12.3 | 5 | 21 | 1-35 |
| TAS-ET | 19 | 5.1 | 27 | 1-40 |
| AMI-ES | 1.33 | 0.74 | 4 | 0.0-4.0 |
| AMI-BA | 1.4 | 0.87 | 4 | 0.0-4.0 |
| AMI-SM | 2.09 | 0.88 | 4 | 0.0-4.0 |
| IRI-FT | 23.7 | 6.4 | 28 | 1-35 |
| IRI-EC | 26.2 | 6.3 | 28 | 1-35 |
| IRI-PT | 25.8 | 6.1 | 28 | 1-35 |
| IRI-PD | 16.7 | 6 | 28 | 1-35 |

**Table S2. CCA results on proportions of choice**

|  | Weights | Loadings | Cross-Loadings |
| --- | --- | --- | --- |
| SAPAS | 0.384 | -0.141 | -0.059 |
| MSI-BPD | -0.294 | 0.095 | 0.039 |
| SRP-I | 0.164 | -0.262 | -0.109 |
| SRP-Aff | 0.374 | -0.366 | -0.152 |
| SRP-L | -0.174 | -0.166 | -0.069 |
| SRP-Ant | -0.453 | -0.08 | -0.033 |
| DASS-Dep | -0.232 | 0.119 | 0.049 |
| DASS-Anx | 0.336 | -0.03 | -0.012 |
| TAS-DF | -0.638 | 0.141 | 0.059 |
| TAS-IF | 0.292 | 0.01 | 0.004 |
| TAS-ET | 0.438 | -0.345 | -0.143 |
| AMI-ES | 0.557 | -0.502 | -0.208 |
| AMI-BA | -0.418 | 0.183 | 0.076 |
| AMI-SM | -0.203 | 0.136 | 0.057 |
| IRI-FT | 0.293 | 0.003 | 0.001 |
| IRI-EC | -0.247 | 0.428 | 0.178 |
| IRI-PT | 0.048 | 0.328 | 0.136 |
| IRI-PD | -0.306 | -0.075 | -0.031 |

**Table S3. Commonality analysis on proportions of choice**

|  | Unique | Common | Total |
| --- | --- | --- | --- |
| SAPAS | .0048 | -.0014 | .0034 |
| SRP-Aff | .0090 | .0140 | .0230 |
| SRP-Ant | .0146 | -.0135 | .0011 |
| DASS-Anx | .0028 | -.0027 | .0002 |
| TAS-DF | .0304 | -.0269 | .0034 |
| TAS-ET | .0159 | .0045 | .0204 |
| AMI-ES | .0236 | .0196 | .0433 |
| AMI-SM | .0270 | -.0212 | .0058 |
| IRI-EC | .0009 | .0306 | .0315 |
| IRI-PT | .0000 | .0185 | .0185 |
| IRI-PD | .0133 | -.0123 | .0010 |
